# Supplementary figures and images for: Machine Learning to Quantify In Situ Humoral Selection in Human Lupus Tubulointerstitial Inflammation
Source: Front Immunol. 2020 Nov 27;11:593177. doi: 10.3389/fimmu.2020.593177 (PMC7731665; doi:10.3389/fimmu.2020.593177)

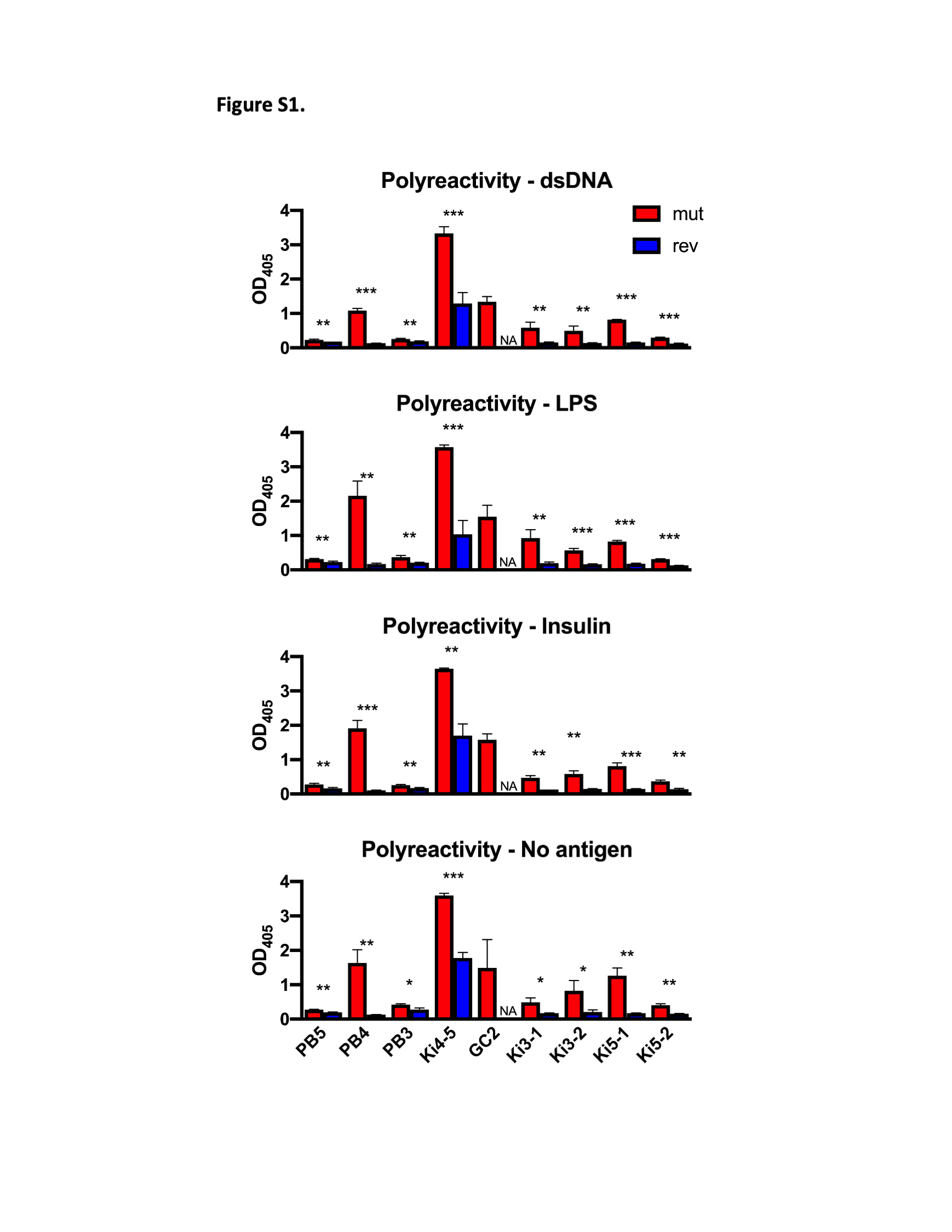

Supplement: Supplementary Figure 1 — ELISAs for polyreactivity. Reactivity of AVAs (mut and rev) with dsDNA, insulin, LPS, or uncoated plates was measured by ELISA (Raw OD405 values are given and t-tests were performed). *q < 0.05, **q < 0.001, ***q < 0.001. [file DataSheet_1.zip › Supplementary Figure S1.TIFF]

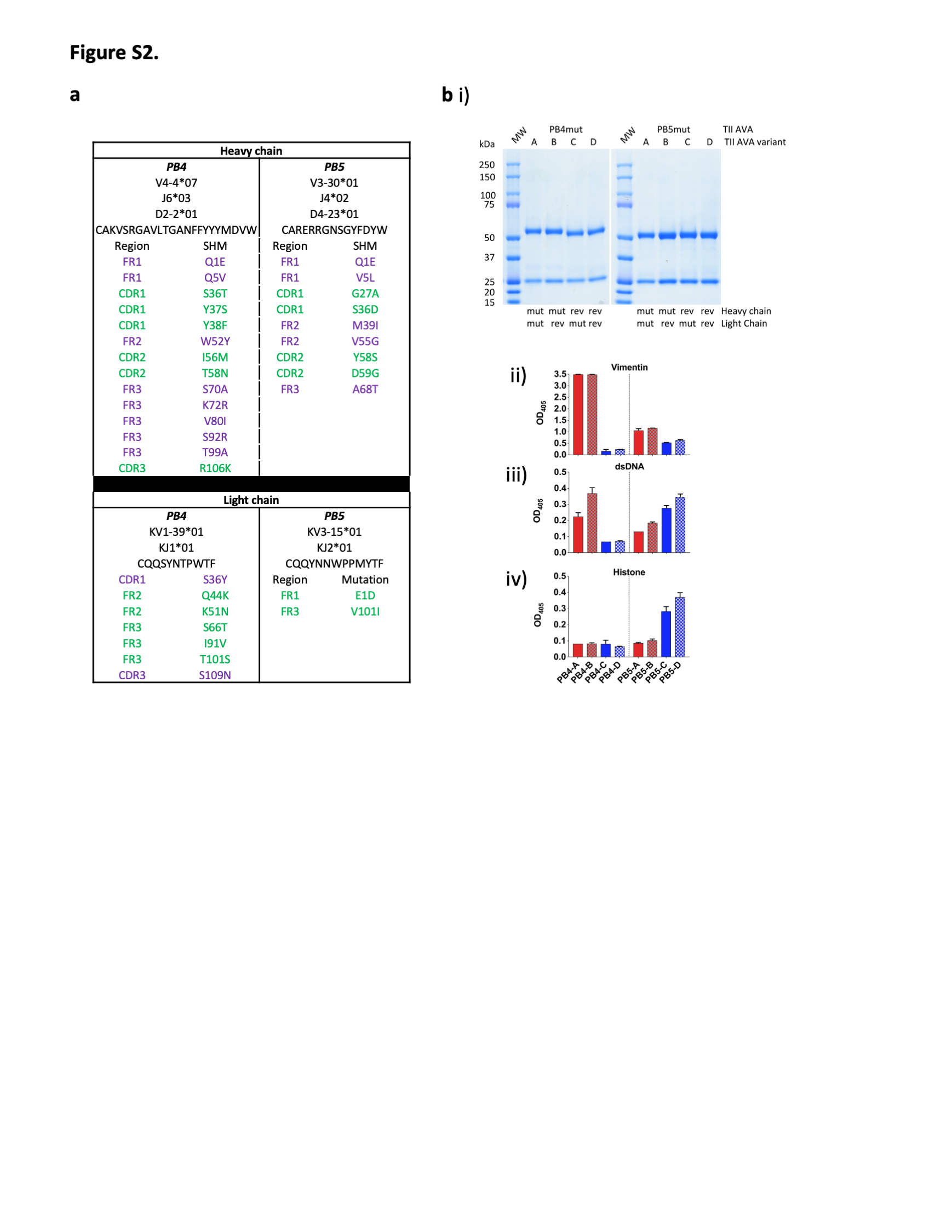

Supplement: Supplementary Figure 1 — ELISAs for polyreactivity. Reactivity of AVAs (mut and rev) with dsDNA, insulin, LPS, or uncoated plates was measured by ELISA (Raw OD405 values are given and t-tests were performed). *q < 0.05, **q < 0.001, ***q < 0.001. [file DataSheet_1.zip › Supplementary Figure S2.TIFF]

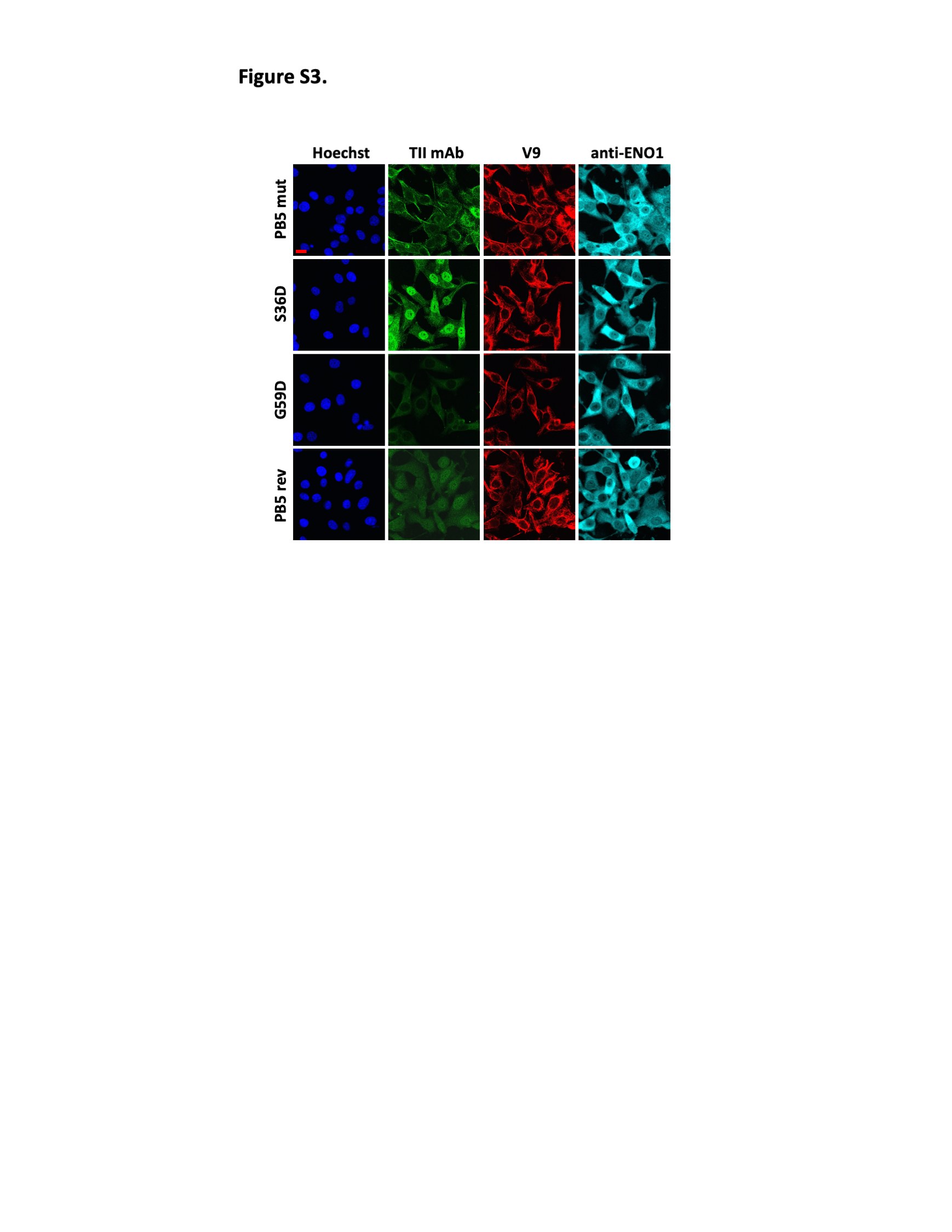

Supplement: Supplementary Figure 1 — ELISAs for polyreactivity. Reactivity of AVAs (mut and rev) with dsDNA, insulin, LPS, or uncoated plates was measured by ELISA (Raw OD405 values are given and t-tests were performed). *q < 0.05, **q < 0.001, ***q < 0.001. [file DataSheet_1.zip › Supplementary Figure S3.TIFF]

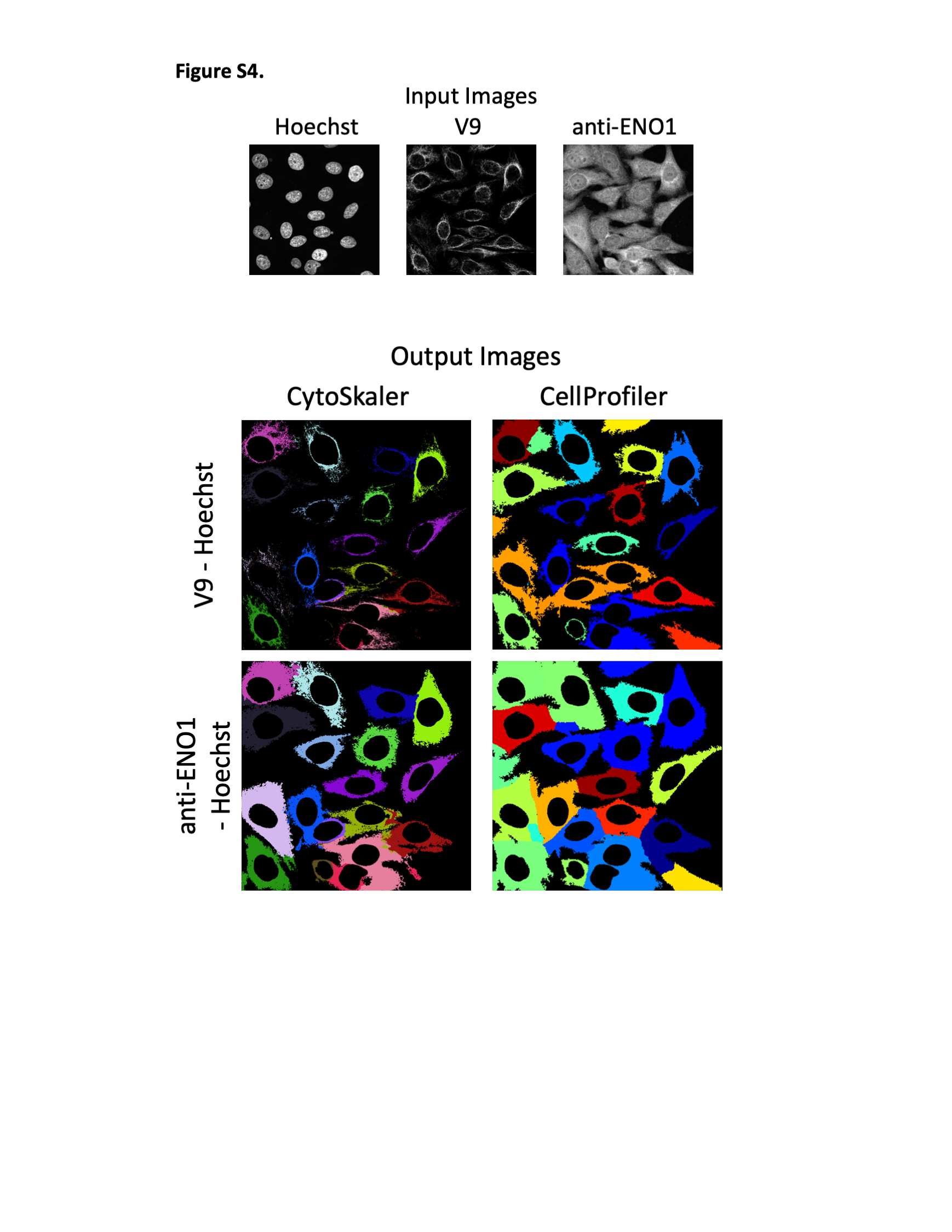

Supplement: Supplementary Figure 1 — ELISAs for polyreactivity. Reactivity of AVAs (mut and rev) with dsDNA, insulin, LPS, or uncoated plates was measured by ELISA (Raw OD405 values are given and t-tests were performed). *q < 0.05, **q < 0.001, ***q < 0.001. [file DataSheet_1.zip › Supplementary Figure S4.TIFF]
