# Supplementary material for: Machine Learning to Quantify In Situ Humoral Selection in Human Lupus Tubulointerstitial Inflammation
Source: Front Immunol. 2020 Nov 27;11:593177. doi: 10.3389/fimmu.2020.593177 (PMC7731665; doi:10.3389/fimmu.2020.593177)
Supplement: Supplementary Table 2 — Equilibrium dissociation constants (KDs) of AVA TII antibodies. [file Table_2.docx]

| **Supplemental Table 2: KDs of TII AVAs** | |
| --- | --- |
| *mAb* | *Kd* |
| PB4 | 3.578E-08 |
| PB4rev | low |
| PB5 | 5.06333E-07 |
| PB5rev | 8.67667E-07 |
| Ki3-1 | 2.846E-07 |
| Ki3-1rev | 5.358E-07 |
| Ki3-2 | 9.652E-08 |
| Ki3-2rev | 1.974E-07 |
| Ki4-5 | 6.817E-08 |
| Ki4-5rev | 2.1005E-07 |
| Ki5-1 | 5.349E-07 |
| Ki5-1rev | low |
| Ki5-2 | 4.208E-07 |
| Ki5-2rev | low |
| PB3 | 0.000001059 |
| PB3rev | low |
| GC2 | 0.000002246 |
| “low”=mAbs with too little reactivity for Kd calculation | |
